# Method to Alleviate Dilutional Coagulopathy Caused by Continuous Renal Replacement Therapy Introduction in a Low-Birth-Weight Neonate: A Case Report

**DOI:** 10.7759/cureus.39556

**Published:** 2023-05-27

**Authors:** Satoshi Nakajima, Kentaro Ide, Emily Knaup, Shotaro Matsumoto, Satoshi Nakagawa

**Affiliations:** 1 Critical Care Medicine, National Center for Child Health and Development, Tokyo, JPN

**Keywords:** low birth weight infant, hemodialysis complication, thrombocytopenia, fresh frozen plasma (ffp), acquired coagulopathy, children, continuous renal replacement therapy (crrt)

## Abstract

Continuous renal replacement therapy (CRRT) in neonates and children has recently been used to treat hyperammonemia and metabolic disorders. However, CRRT introduction in low-birth-weight neonates is still a challenge due to vascular access limitations, bleeding complications, and a lack of neonatal-specific devices. We present the case of a low-birth-weight neonate whose severe coagulopathy due to CRRT introduction with a red cell concentration-primed circuit was alleviated by priming the new circuit with blood from the current circuit. This male preterm infant (birth weight: 1,935 g) was admitted to the pediatric intensive care unit at two days old with metabolic acidosis and hyperammonemia, which required CRRT. Following CRRT introduction, he showed marked thrombocytopenia (platelet count: 305,000-59,000/μL) and coagulopathy (prothrombin time international normalized ratio (PT/INR) >10), necessitating platelet and fresh frozen plasma transfusions. Upon circuit exchange, we primed the new circuit with blood from the current circuit. This resulted in only a slight worsening of thrombocytopenia (platelet count: 56,000-32,000/μL) and almost no change in coagulation (PT/INR: 1.42-1.54). We also reviewed the literature regarding safe CRRT management in low-birth-weight neonates. Since there is no established method for the use of blood from the current circuit during circuit exchange, this should be addressed in future work.

## Introduction

Continuous renal replacement therapy (CRRT) in neonates and children has recently been identified as an important treatment for hyperammonemia and metabolic disorders [1,2]. However, large extracorporeal circuit volumes are particularly problematic in low-birth-weight neonates because the priming volume (PV) of the circuit is relatively large compared with their blood volume [3]. Thus, when the PV is >10% of the patient’s blood volume, priming the circuit with red cell concentrate (RCC) is recommended to avoid complications due to CRRT introduction, such as hypotension or dilutional anemia [4,5]. We experienced the case of a low-birth-weight neonate with severe dilutional coagulopathy due to CRRT introduction with an RCC-primed circuit, whose condition was alleviated by priming the new circuit with blood from the current circuit. We also present a literature review to promote safe CRRT management in low-birth-weight neonates.

## Case presentation

A low-birth-weight (1,935 g) male infant was born prematurely (34 weeks) due to premature rupture of membranes. The next day, he was intubated for neonatal respiratory distress syndrome. On the following day, laboratory tests revealed metabolic acidosis (pH 7.294) and elevated ammonia (NH3) (>500 μg/dL). He was transferred to our pediatric intensive care unit (PICU). On admission, his respiration and circulation were stable, but laboratory tests showed worsening hyperammonemia (994 μg/dL).

**Figure 1:**
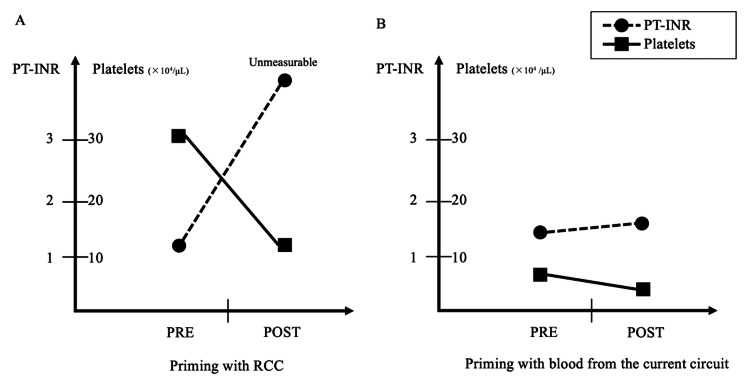
Comparison of PT/INR and platelet count before and after CRRT introduction. Laboratory tests were performed pre-CRRT (one to eight hours before CRRT introduction) and post-CRRT (after 12 hours of CRRT introduction). A. Priming with RCC. B. Priming with blood from the current circuit. PT/INR: prothrombin time international normalized ratio; CRRT: continuous renal replacement therapy; RCC: red cell concentrate

We started intravenous administration of arginine, sodium phenylacetate, sodium benzoate, and vitamins and inserted a 6.0 Fr double-lumen dialysis catheter (BabyFlow; NIPRO, Osaka, Japan) in the right internal jugular vein at the junction of the superior vena cava and right atrium. CRRT was implemented using a continuous renal replacement machine (TR-55X; Toray Medical, Tokyo, Japan), an extracorporeal circuit (U-520SY; Toray Medical; PV: 67 mL), and a cellulose triacetate membrane filter with a surface area of 0.5 m2 (UT filter; NIPRO; PV: 40 mL). We primed the circuit and the membrane filter using 60 mL of RCC and continuously infused nafamostat mesilate (Torii Pharmaceutical, Tokyo, Japan) into the circuit as a local anticoagulant. CRRT was performed at a blood flow rate of 20 mL/min (10 mL/kg/min) and a dialysate flow rate of 2,000 mL/hr (1000 mL/kg/hr). After CRRT introduction, the patient became hypotensive (43/23 mmHg) and was started on continuous norepinephrine with a maximum dose of 0.1 µg/kg/min. Although his NH3 level decreased (139 μg/dL) after 12 hours of CRRT, marked thrombocytopenia (platelet count: 305,000-59,000/μL) and coagulopathy (prothrombin time international normalized ratio (PT/INR)>10) necessitated the transfusion of platelets and fresh frozen plasma (FFP) (Figure 1).

After CRRT introduction, NH3 levels were maintained < 150 µg/dL. On day two of PICU admission, we changed the CRRT circuit and preemptively transfused platelets and FFP to prevent thrombocytopenia and coagulopathy. For the second circuit exchange, on day five of PICU admission, we primed the circuit with blood from the current circuit. The new saline-primed circuit was placed in a closed loop, and the blood pump was set at a flow rate of 100 mL/min. Then, we stopped the current circuit and removed it from the patient, connected the tip of the arterial side of the current circuit to the new circuit, and set the blood pump of the current circuit at a flow rate of 20 mL/min (the venous side was open to the air). At the same time, we started the drainage pump of the new circuit at a rate of 1,200 mL/hr to maintain the pressure in the new circuit, where the blood from the current circuit was supplied at 20 mL/kg/min (1,200 mL/hr). The blood in the current circuit (107 mL) was primed into the new circuit in approximately five minutes. There was no episode of hypotension during the introduction. Furthermore, there was only a slight worsening of thrombocytopenia (platelet count: 56,000-32,000) but almost no change in coagulation (PT/INR: 1.42-1.54) (Figure 1) and anemic status (hemoglobin: 11.6-11.1 mg/dL). The patient was weaned off CRRT on day eight, and hyperammonemia did not reoccur. He was diagnosed with transient neonatal hyperammonemia. He underwent a tracheotomy for central hypoventilation on day 41 and was transferred to the general ward on day 48.

## Discussion

We experienced a case of a low-birth-weight neonate whose severe dilutional coagulopathy due to CRRT introduction with an RCC-primed circuit was alleviated by priming the new circuit with blood from the current circuit. There are several reports on using blood from the current circuit when introducing CRRT to prevent complications such as hypotension or dilutive anemia. However, to our knowledge, this is the first report on the effect of this method on coagulopathy.

**Figure 2:**
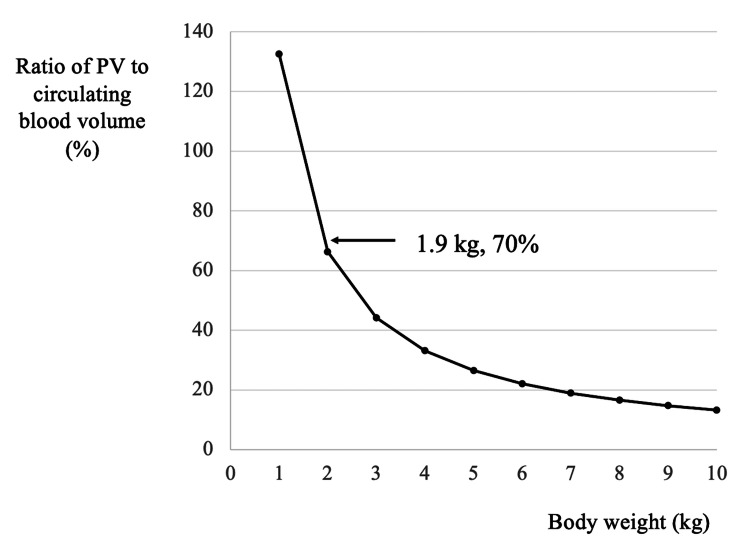
Relationship between the patient’s body weight (X-axis) and the ratio of PV to circulating blood volume (Y-axis). Circulating blood volume was estimated at 8% of body weight. PV: priming volume

The use of blood from the current circuit when introducing CRRT has been reported as safe and effective [6-8]. Yorgin et al. reported a priming method that transferred blood from the current circuit to the new circuit connected in series [6]. They utilized this method more than 30 times for at least 15 children weighing <15 kg. Since no adverse events were observed, they concluded that it might benefit children with hemodynamic or clinical instability. Eding et al. reported a circuit-to-circuit exchange method that transferred blood from the patient to a new circuit while simultaneously returning blood from the current circuit to the patient [7]. They utilized this method for 12 children (aged four days to eight years old) for a total of 20 exchanges and concluded that their method was an effective transition technique from a current to a new circuit. In our case, the use of blood from the current circuit alleviated not only the patient’s hypotension and anemia but also his coagulopathy. Figure 2 shows a graph of the relationship between a patient’s body weight and the ratio of PV to circulating blood volume (estimated at 8% of body weight). The ratio of PV to circulating blood volume is approximately 44% at a body weight of 3 kg but rises sharply to 70% at a body weight of 1.9 kg. Thus, patients weighing <3 kg may be prone to dilutional coagulopathy, and we suggest administering FFP during the initial introduction (including FFP priming) or using blood from the current circuit during circuit exchange in low-birth-weight neonates.

Although the use of blood from the current circuit is beneficial to patients, there are several issues and limitations that were experienced. First, there is no established method for the use of blood from the current circuit. Yorgin et al. primed the new circuit with blood from the current circuit, as did we [6]. However, Eding et al. and Zhou et al. returned blood from the current circuit to the patient while transferring blood from the patient to a new circuit [7,8]. Thus, further research is necessary to establish a method for safe implementation. Second, the method requires several trained personnel to operate and monitor two dialysis machines, and performance by inexperienced staff may lead to prolonged stasis of blood in the pathway, resulting in clotting and loss of the circuit [6]. Third, the method needs two dialysis machines, which require a considerable amount of bedside space. Thus, both equipment availability and bedside space can limit the usefulness of the method because the two dialysis machines must be placed next to each other, each with access to the dialysis catheter [6,7]. Fourth, the method has yet to be replicated using different brands of dialysis machines. Although it is likely that other dialysis machines will be equally effective, validation of the method with other dialysis machines and in other settings is warranted [7]. Fifth, the method must be performed while the current circuit is still in a functioning state [6,7]. Thus, preventive circuit exchange is required to avoid the current circuit becoming nonfunctional due to clotting, and this may increase the frequency of circuit exchange.

## Conclusions

We experienced a case of a low-birth-weight neonate with severe coagulopathy due to CRRT introduction with an RCC-primed circuit. His condition was alleviated by priming the new circuit with blood from the current circuit. The use of blood from the current circuit is beneficial for preventing not only hypotension and RCC transfusions but also FFP transfusions. However, further research is crucial to establishing a method for safe implementation.
